# On the thermal effect induced in tissue samples exposed to extremely low-frequency electromagnetic field

**DOI:** 10.1186/s40201-015-0241-8

**Published:** 2015-12-17

**Authors:** M. Racuciu, S. Miclaus, D. Creanga

**Affiliations:** Environmental Sciences and Physics Department, Faculty of Sciences in “Lucian Blaga” University, Dr. I. Ratiu Street, no. 5-7, Sibiu, 550024 Romania; Technical Sciences Department, ”Nicolae Balcescu” Land Forces Academy, Revolutiei Street, no. 3-5, Sibiu, 550170 Romania; Biophysics and Medical Physics Laboratory, Faculty of Physics, “Alexandru Ioan Cuza” University, 11, Blvd. Carol I, Iasi, 700506 Romania

**Keywords:** Extremely low frequency electromagnetic field, Heating dynamics effect, Mammalian tissues

## Abstract

**Background:**

The influence of electromagnetic exposure on mammalian tissues was approached as a public health issue aiming to reveal the putative side effect of 50 Hz industrial and domestic supply source (i) during aliments storage near such sources; (ii) in people staying couple of hours in the proximity of conducting wires.

**Materials and methods:**

Fluorescence emission based thermal sensor was used to emphasize temperature dynamics of fresh meat samples during controlled electromagnetic exposure in Helmholtz coils adjusted to deliver 50 Hz / (4÷10) mT electromagnetic field in their inner volume. Fluoroptic temperature probe with 0.1 °C accuracy measurement and data acquisition software allowed reading temperature every second, in the tissue volume during exposure.

**Results:**

The temperature dynamics curves of *ex-vivo* porcine tissues like liver, kidney, brain, muscle, lung, and bone, were comparatively analyzed – the choosing of the mammalian species being justified by metabolic and physiological similarities with human body. The curve slopes appear to be the same for the range of initial temperatures chosen to perform the tests (20.0 ± 0.1 °C), the temperature increase reaching around 2.0 °C for the magnetic flux density of 10 mT. Quantitative dependence was evidenced between the thermal effect and the magnetic flux density.

**Conclusions:**

The technical interpretation is based on heating effect, on bioimpedance increasing and on water vaporization during wet sample exposure. The biomedical aspects derive from the degrading effects of food heating as well as from possible *in vivo* effects of living body exposure.

## Background

During last decades there has been an increasing interest in the bioeffects of the electromagnetic fields interaction with living organisms, with focus on potential health hazard. The biological effects of industrial alternative current with 50 Hz standard frequency have been much discussed in the context of the biological response to extremely low frequency magnetic field (ELF-MF). All living organisms are continuously exposed to electromagnetic fields from industrial and domestic sources. It seems clear now that electromagnetic exposure can induce biological changes, although the precise effects are not yet well known. In recent decades, many scientific studies have confirmed that magnetic fields of extremely low frequency (ELF; frequency <300 Hz) can influence the biological systems. Data reported in the literature regarding direct effects induced by ELF-MF on cell functions are controversial and the interaction mechanisms of electromagnetic fields with biological systems are still partially understood [1].

The hypothesis that electromagnetic field may act as initiator or co-initiator of carcinogen tumours [2, 3] underlied the 1999 decision of U.S. National Institute of Environmental Health Sciences to include electromagnetic fields in the category of “possible human carcinogen”. Also, in [4] it was reported the increasing of cancer rates in children when exposed at home at 50Hz magnetic fields greater than 0.3 μT. The bioeffects of environmental constraints of electromagnetic nature are very difficult to explain since living bodies are complex dynamic systems, with many physical and chemical parameters non-homogeneously distributed and time dependent. Therefore the assumption on non-thermal mechanisms of living organism electromagnetic exposure in the lack of any relationship with general or localized temperature increase with a consequent thermal distortion of bio-molecules still remains a scientific challenge. Actually thermal effects of electromagnetic field, especially low subtle heating effects are still considered the main cause of biological damages when the focus is the major role of thermal sensitivity of enzyme catalytic activity. That is why homeostatic processes from living cells and tissues could be perturbed by couple degrees temperature rise and such small perturbation could damage the whole organism when amplify at larger scale of space and time. Injuries of cell biochemistry triggered following human electromagnetic exposure could be an actual concern for public health, biomedicine [5, 6] and food science. In contrast with radiofrequency region of the spectrum, in the ELF region, direct thermal effect in tissues was neither definitely revealed nor sufficiently analyzed up-to-now – according to International Commission on Non-Ionizing Radiation Protection [7].

Having in mind that water is the dominant element hosting all biomolecules from living matter, an unusual phenomenon of weak ELF magnetic field impact on water should underline the reported bioeffects as long as the field energy is much lower than hydrogen bonds characterizing water molecule organization; but for now, an acknowledged mechanism of influence of weak ELF magnetic field on water does not exist. Some researchers had observed the changes of water electric conductivity parameters due to action of a weak ELF magnetic field (f ≤ 50 Hz) [8]. In [9] the authors have shown the increasing of the water evaporation rate due to the exposure to weak static magnetic fields (15 mT). Other studies led to controversial results.

In [10] the authors reported that, using a thermocouple with a precision of 0.1 °C no changes in temperature were detected for 915 MHz or 50 Hz exposures of human lymphocytes culture. Other researchers [11] reported that heat shock protein synthesis in cells exposed to 50 Hz/(0–100 μT) at 40 °C was not increased compared to cells incubated at 40 °C without magnetic field exposure. In [12] it was studied the exposure of endothelial cells culture to domestic power supply (50 Hz/700 μT) that resulted in no detectable effects on the expression of heat shock protein60 as the dominant autoantigen in endothelial cells. Thermal effect of 50 Hz/94 mT magnetic field was only reported in inert materials such as superconductors [13].

Some other reports appear to evidence opposite results such as those regarding cell apoptosis after electromagnetic exposure. So, exposure of human lymphocytes at room temperature to either 915 MHz or 50 Hz resulted in significant condensation of chromatin, as measured through the method of anomalous viscosity time dependencies [10] but no apoptosis induced by DNA morphological changes or by its fragmentation was evidenced; while electromagnetic exposure to 50 Hz/0.097 T was found able to induce and promote apoptosis of mice murine liver cells in time-effects manner as shown in [14].

In [15] it was reported the ohmic heating rate of peaches for electric pulses with frequencies varying from 50 Hz to 1 MHz, thermal damage of tissue being evaluated from electrical admittance; it was found that samples exposed to low-frequency electric field demonstrated faster electro-thermal damage rates. Apart from experimental measurements, mathematical investigations also offered interesting approaches; the theoretical analysis of tissue heating as a potential side effect of strong electric pulses, developed in [16], revealed localized tissue heating near the electrodes which is assessed mainly to the sharp radial decrease of the electric field around the needles.

The main interaction mechanism of low frequency electromagnetic fields with absorbent matter is supposed to be the Faraday induction of electric fields and associated currents, the distribution of the induced electric field depending on the conductivity of organs and tissues [7]. The maximum electric field is induced in the body when the external fields are homogeneous and directed parallel to the body axis or perpendicular to it. According to calculations on human body models [7], the maximum local peak of electric field induced by a 50 Hz magnetic field in the brain is approximately of 23–33 mV/m per mT, depending on field orientation and body model. The corresponding electric field induced in the skin is of approximately 20–60 mV/m per mT.

While most of the experiments already mentioned were focused on the *in vivo* electromagnetic exposure there is less literature regarding the effects induced in tissue samples exposed immediately after excision from animal body (*ex vivo* exposure) – which would be of interest for food processing and technology rather than for bio-electromagnetism.

Our study was designed to evidence putative effects of electromagnetic exposure in fresh food samples with practical importance for temporal food storing near 50 Hz supplied devices. The experimental work was focused on the measurement of thermal effect dynamics in *ex vivo* biological tissues (during couple of hours following withdrawn from animal body) when exposed to 50 Hz electromagnetic field (4 mT-10 mT magnetic flux density). The heating was recorded during about 3,000 s of continuous electromagnetic exposure.

## Methods

### Biological material

Mammalian tissue samples were consistent with specimens of porcine liver, kidney, lung, brain, muscle and bone, freshly excised, each of approximately 1 cm^3^ – as resulted from direct volumetric estimation. Porcine tissue samples considered for the study had different masses, ranging within 3–4 g, because of their different densities. New tissue sample for every magnetic field exposure was used. Comparatively temperature dynamics was recorded for 1 ml deionized water as well as in the free air around the same point from the Helmholtz coil system centre.

### Electromagnetic exposure

**Fig. 1:**
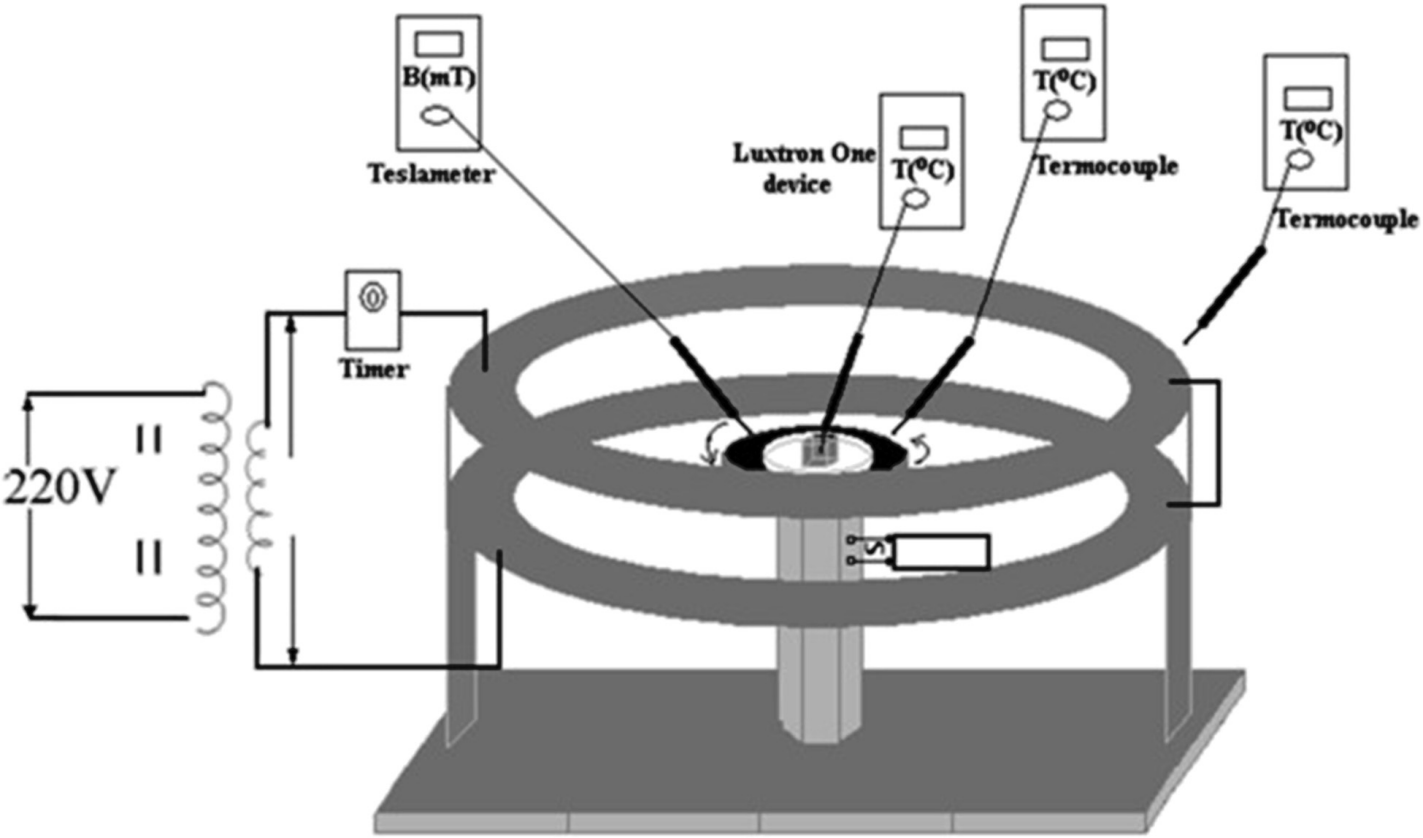
Experimental set-up; coil system supplied by power transformer connected to industrial electricity grid

**Fig. 2:**
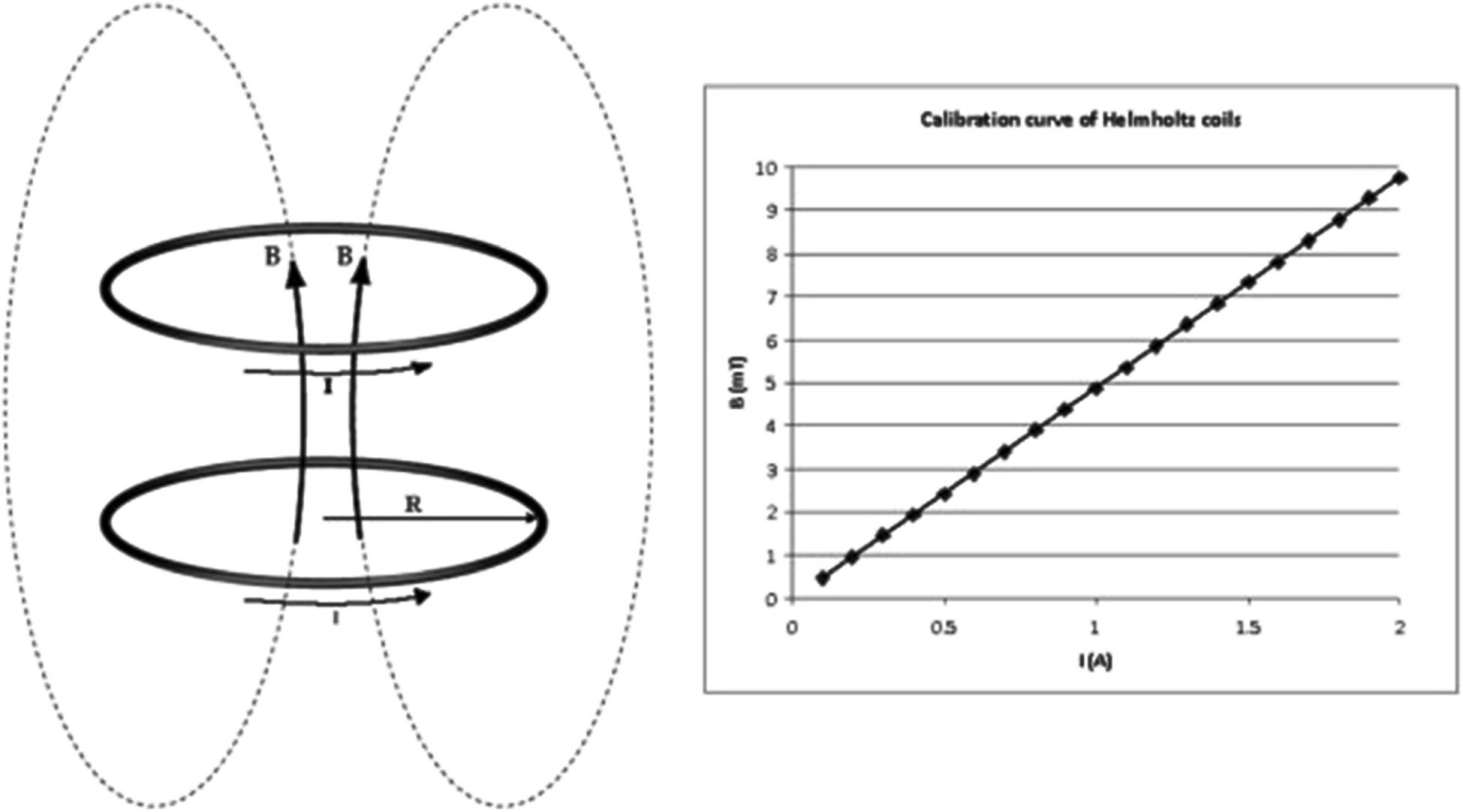
Calibration curve of Helmholtz coil system using a low-frequency field analyzer, NARDA EFA-300

Electromagnetic exposure of tissue specimens was carried out within a Helmholtz coil system (Fig. 1) able to generate a vertical magnetic field of 50 Hz frequency and 4 mT, 6 mT, 8 mT and respectively 10 mT magnetic flux density for current intensity of 0.82 - 1.23 - 1.64 -2.05 A - as measured with a device type Simpson 260 Analog - VOM. Preliminary magnetic field measurements were carried out using a low-frequency field analyzer, NARDA EFA-300, before the mammal tissues magnetic exposure, based on the calibration curve of Helmholtz coil system presented in Fig. 2. The measurements of the magnetic field induction evidenced that within the centre of the Helmholtz coils system no significant variations of the field could be detected within 100 mm diameter area. The Helmholtz coil system consisted of two coils, each formed by 1,000 turns of 1 mm cooper wire, with a mean diameter of 260 mm and a thickness of 25 mm. The coils were mounted coaxially and placed at a mean distance of 130 mm from each other (Figs. 1, 2). The magnetic exposure was done by placing the mammal tissue sample on a glass dish (90 mm diameter), as dielectric support, in the centre of the coil system. The space chosen for the experiment was consistent with a small room arranged as thermostat with electric supply, with no windows - only double wall door; constant air temperature within the working space was displayed on thermocouples continuously during the tissue investigation project. No person was in that room during experimental recordings.

### Temperature measurement

A Luxtron One fiber optic device was used to accomplish temperature recording. This one is provided with a thermal fluoroptic probe, of 1.5 mm diameter and working on the principle of fluorescence quenching in thermo-resistant phosphorescent sensor. The fiber optic probe was inserted inside the mammal tissue, in the centre of volume, through a tiny incision with the size of the probe, made at the time of its insertion. The accuracy of temperature measurement was of ± 0.1 °C, the temperature values being recorded every second for about one hour in each tissue sample and transferred to a PC with TrueTemp3.0 program [17, 18]. Then temperature/time graphs were plotted using Origin 7.5 software.

Repeated recordings were carried out three times on similar samples extracted from each tissue – the bulk sample being kept at refrigerator (4 °C) and let to reach the environmental controlled temperature (of 20 °C) before new aliquot cutting. After thermal investigation any used tissue sample appeared less wet and structurally changed so that new incision and temperature measurement repetition seemed not reliable. Representative data series were presented and discussed below.

## Results and discussions

**Fig. 3:**
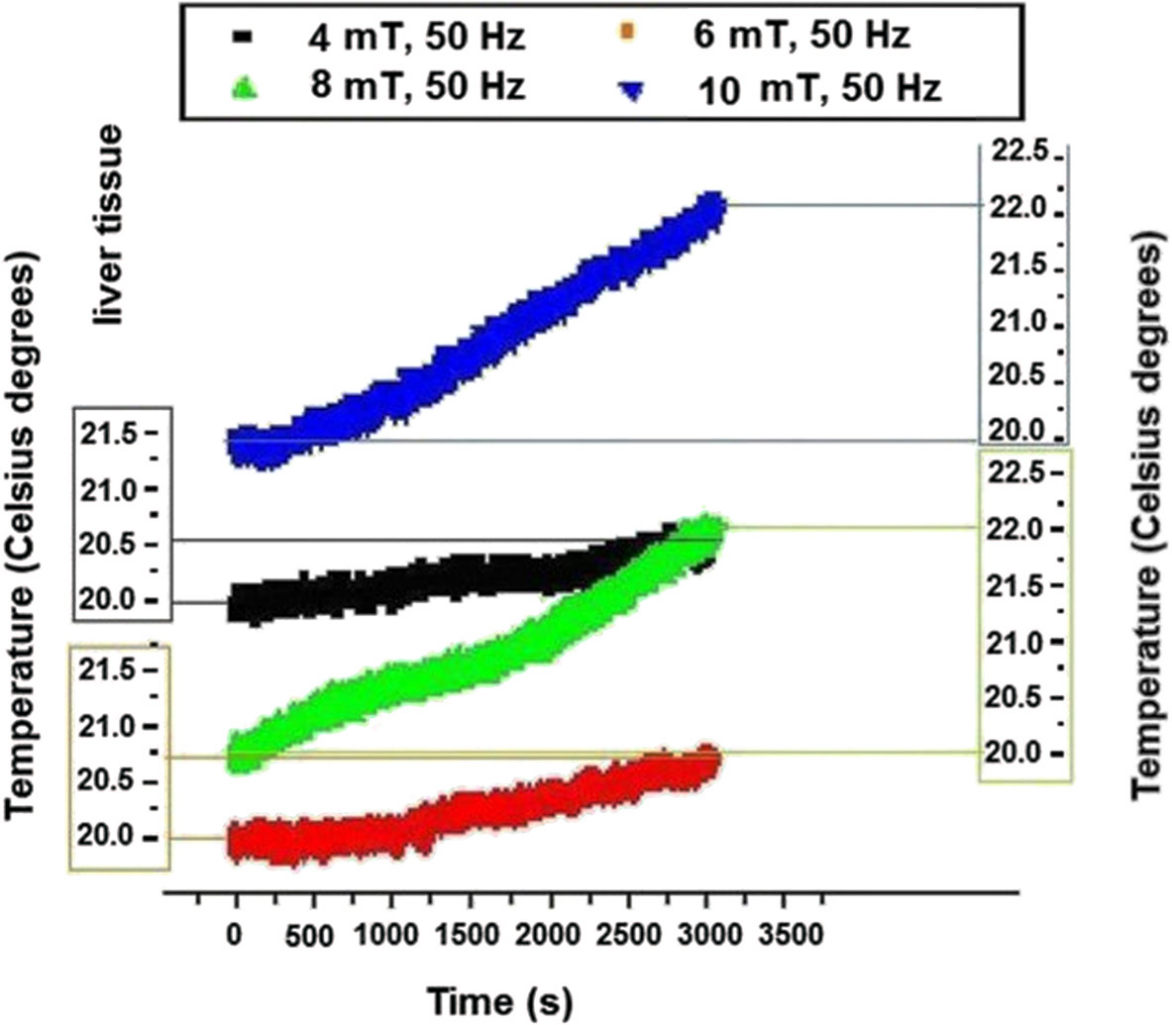
Temperature variation during electromagnetic exposure of liver tissue

The temperature dynamics curves were recorded for each magnetic flux density values of 4-6-8-10 mT starting from the same initial temperature (of 20.0 ± 0.1 °C). In the free air (sample missing) the temperature measured in coil system centre exhibited no variation during 3,000 s. The same occurred in tissue samples when coils system was not electrically supplied. For electromagnetically exposed liver tissue the temperature increase in Fig. 3 is presented. The curves were translated on the ordinate in order to be grouped in tissue family of curves – and this is valuable for all similar plots presented below.

For relatively low magnetic flux density, of 4 mT and 6 mT the temperature increase was of only 0.5 °C and respectively 0.7 °C but for higher magnetic flux density (8 mT, respectively 10 mT) about 1.8 °C and respectively 2.0 °C increases in liver tissue sample temperature were recorded.

**Fig. 4:**
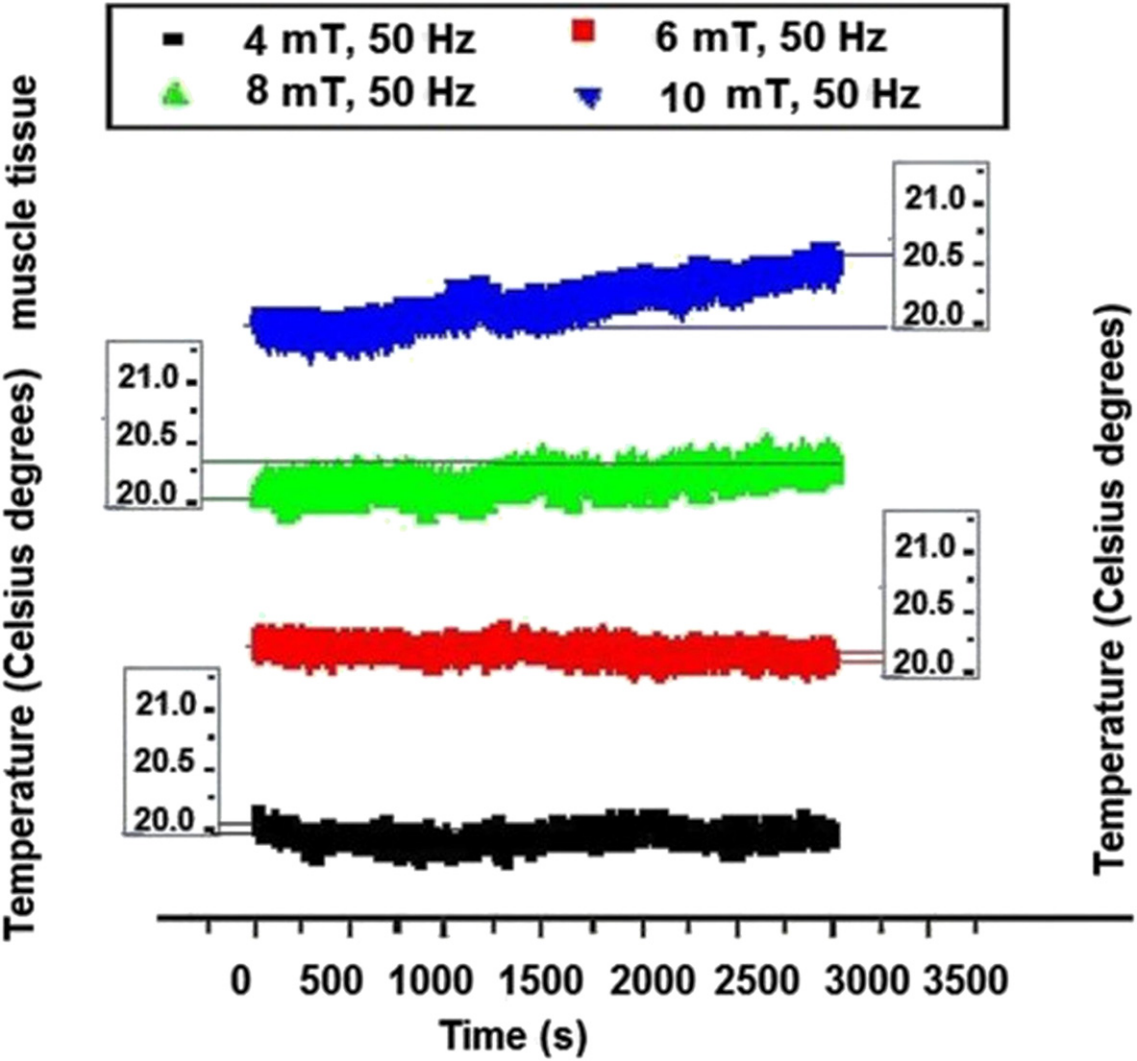
Temperature dynamics in muscle tissue electromagnetically exposed

**Fig. 5:**
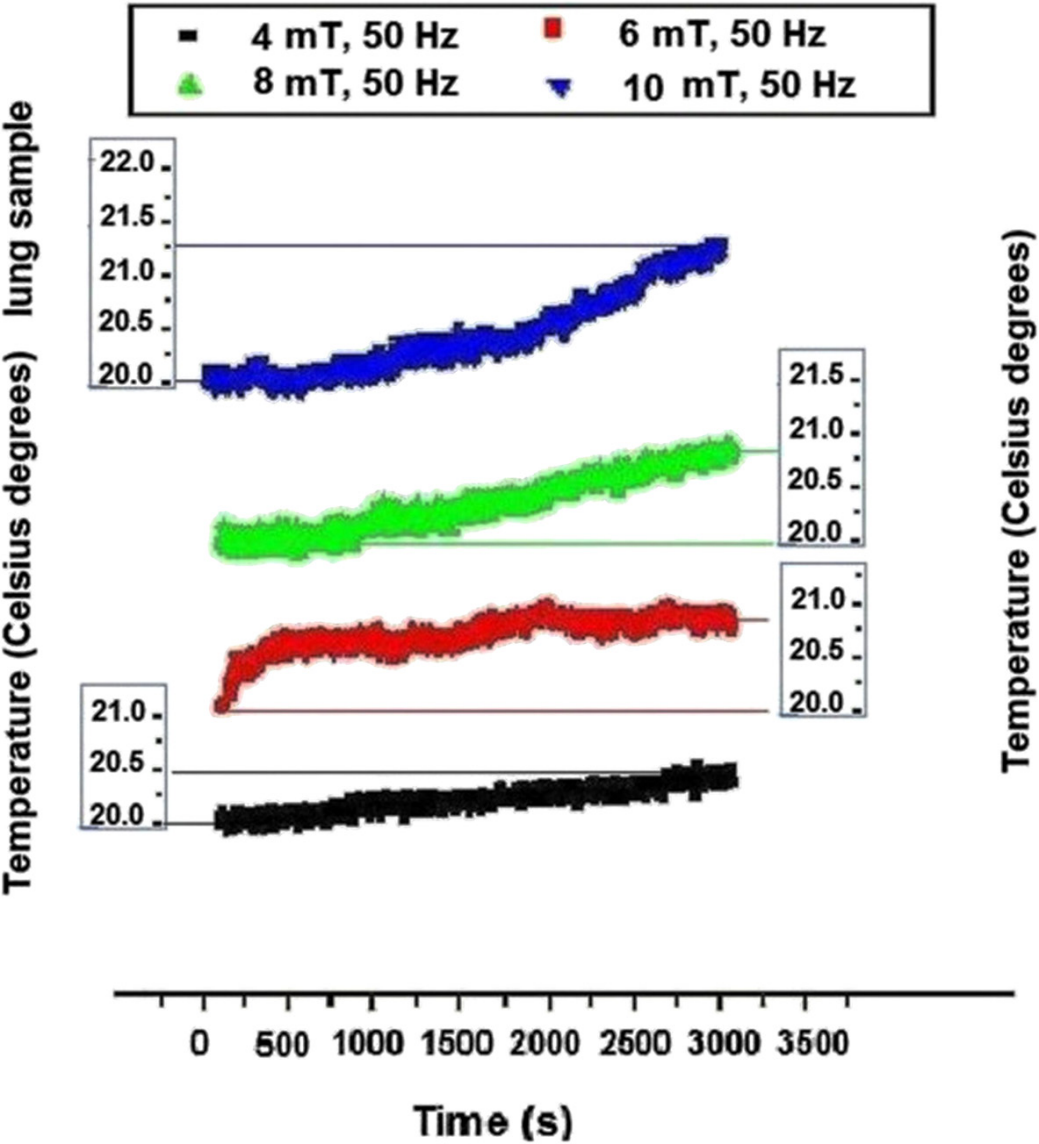
Temperature dynamics in lung tissue sample electromagnetically exposed

Detectable variation of temperature within muscle tissue was evidenced (Fig. 4) only for 8 mT (about 0.3 °C in 3,000 s) and for 10 mT (0.5 °C), this being probably the consequence of the considerable evaporation of water (as muscle is a “wet” tissue) that partially compensated the temperature increase due to the Helmholtz coil system. As well the thermal effect of lowest magnetic flux density applied in this study could be insufficient for inducing detectable heating effect in association also with possible intrinsic peculiarities of muscle tissue. In the next graphs, for the other tissues there was also a slighter heating effect for 4 mT and 6 mT than for 8 mT and 10 mT. In lung tissue (Fig. 5) significant temperature rise was noticed for all magnetic flux densities, from 0.5 °C in the case of 4 mT to 0.7 °C in the case of 6 mT and 0.9 °C in the case of 8 mT with further increase up to 1.2 °C for 10 mT. Certain different variation trend was noticed for 6 mT graph which is probably related to the lung tissue lacunary structure – as further in the bone can be seen.

**Fig. 6:**
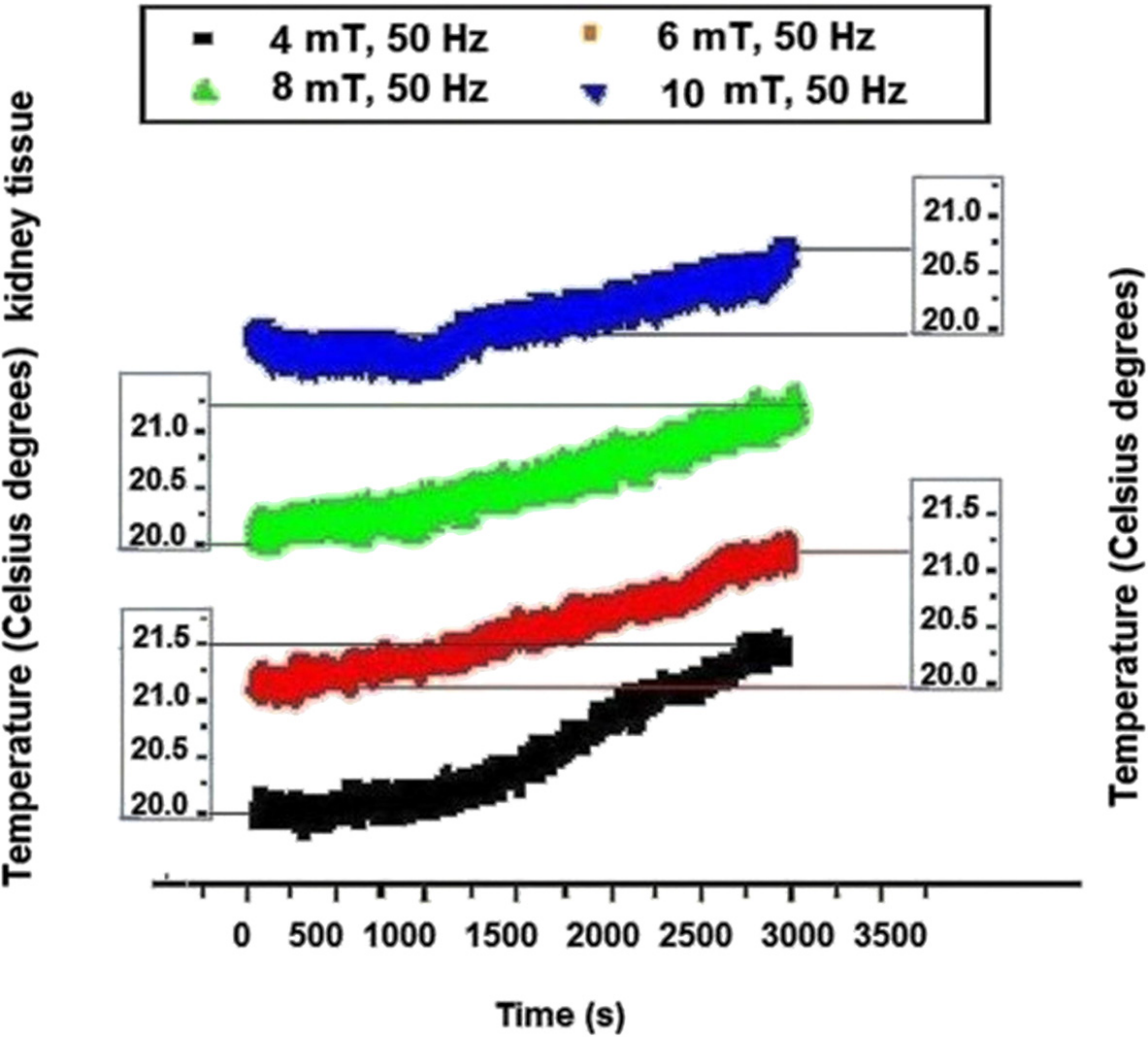
Temperature recording in the electromagnetically exposed kidney sample

In Fig. 6 the temperature measured in kidney tissue is presented where the increase noticed for 4 mT was of about 0.6 °C, the increases for 6 mT and respectively for 8 mT magnetic fields were of about 1.0 °C and 1.1 °C respectively, while finally, for 10 mT the highest 1.3 °C positive variation was recorded.

**Fig. 7:**
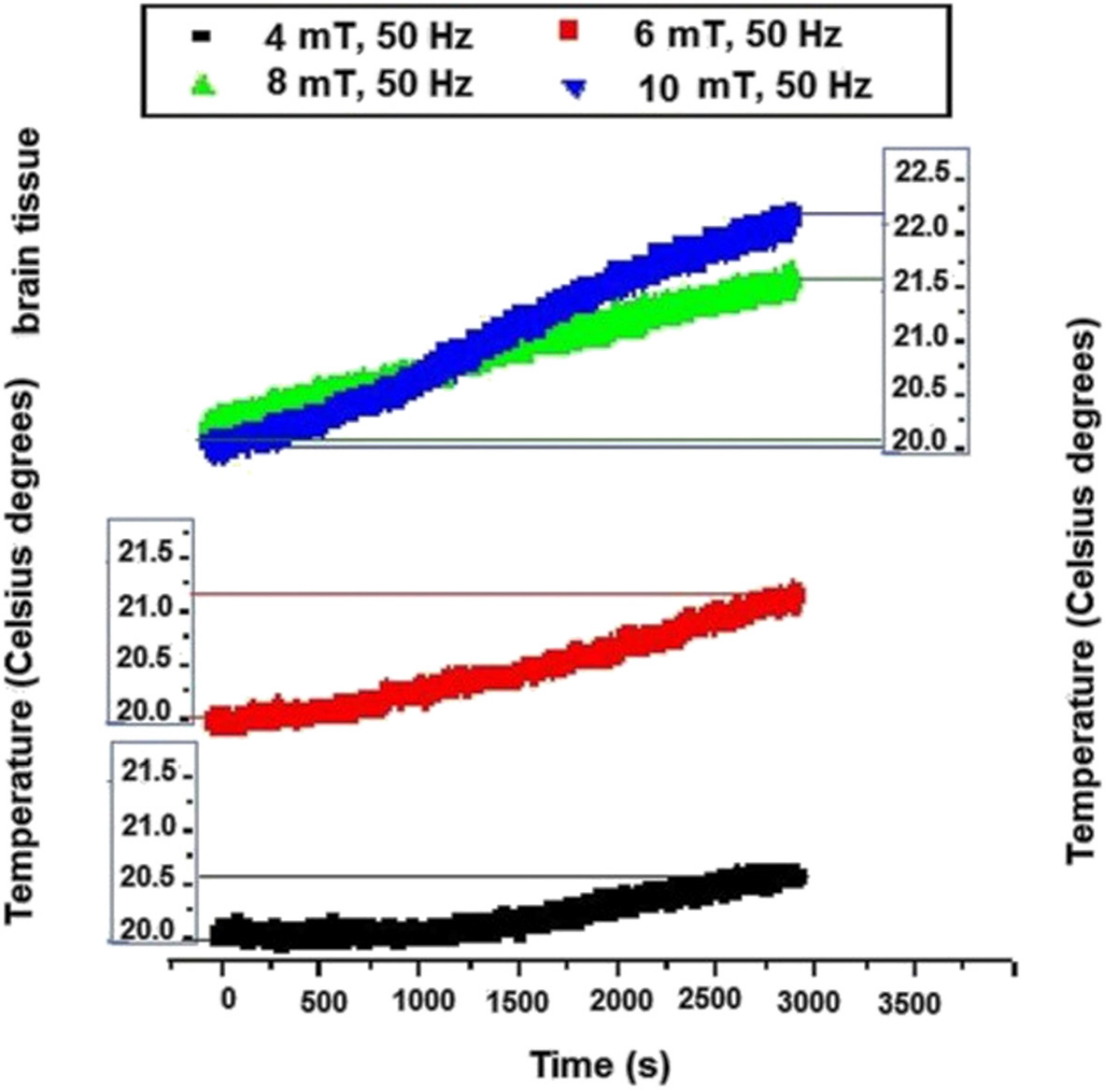
The temperature recording in brain tissue electromagnetically exposed

The data resulted from temperature measurement in brain tissue are given in Fig. 7.

At least 0.5 °C positive variation of temperature was recorded for 4 mT that was further progressively increased for 6 mT (at about 1.2 °C), for 8 mT (1.5 °C) as well as for 10 mT (2.2 °C). This remarkable heating amplitude could be associated with the highest fat content that seems to reduce specific heat capacity of tissues [19].

**Fig. 8:**
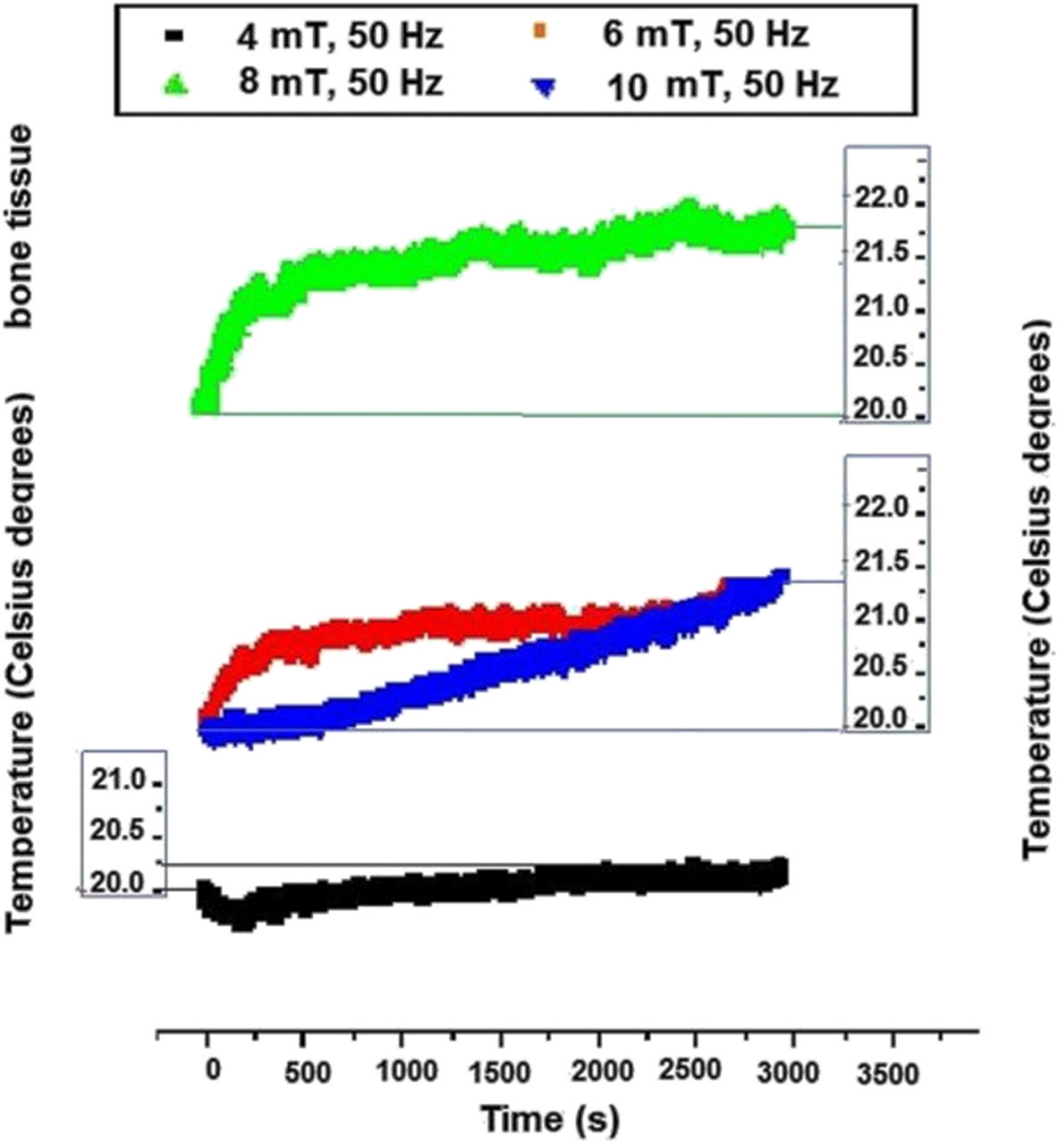
The temperature recording in bone tissue electromagnetically exposed

The response of bone tissue following the exposure to 50 Hz electromagnetic field in Fig. 8 is presented.

The bone tissue responded with only 0.2 °C temperature increasing for 4 mT but the positive temperature variation has increased to about 1.2 °C for 6 mT and 1.5 °C for 8 mT, reaching 1.7 °C increase for 10 mT. Specific shape of the curve first segment, corresponding to first approximately 1,000 s, could be related to the characteristic spongiest bone structure with lowest homogeneity among all tissues; early significant temperature variation during the first part of exposure was followed by saturation tendency up to about 3,000 s. The highest slope was revealed for the highest magnetic induction, i.e. 10 mT, when almost linear graph was recorded with no saturation trend. It could be presumed that 10 mT exposure induced the diminution of thermal conductivity anisotropy in the tissue so that the heat transmission from the environment resulted in the similar trend observed with the other isotropic tissue samples.

**Fig. 9:**
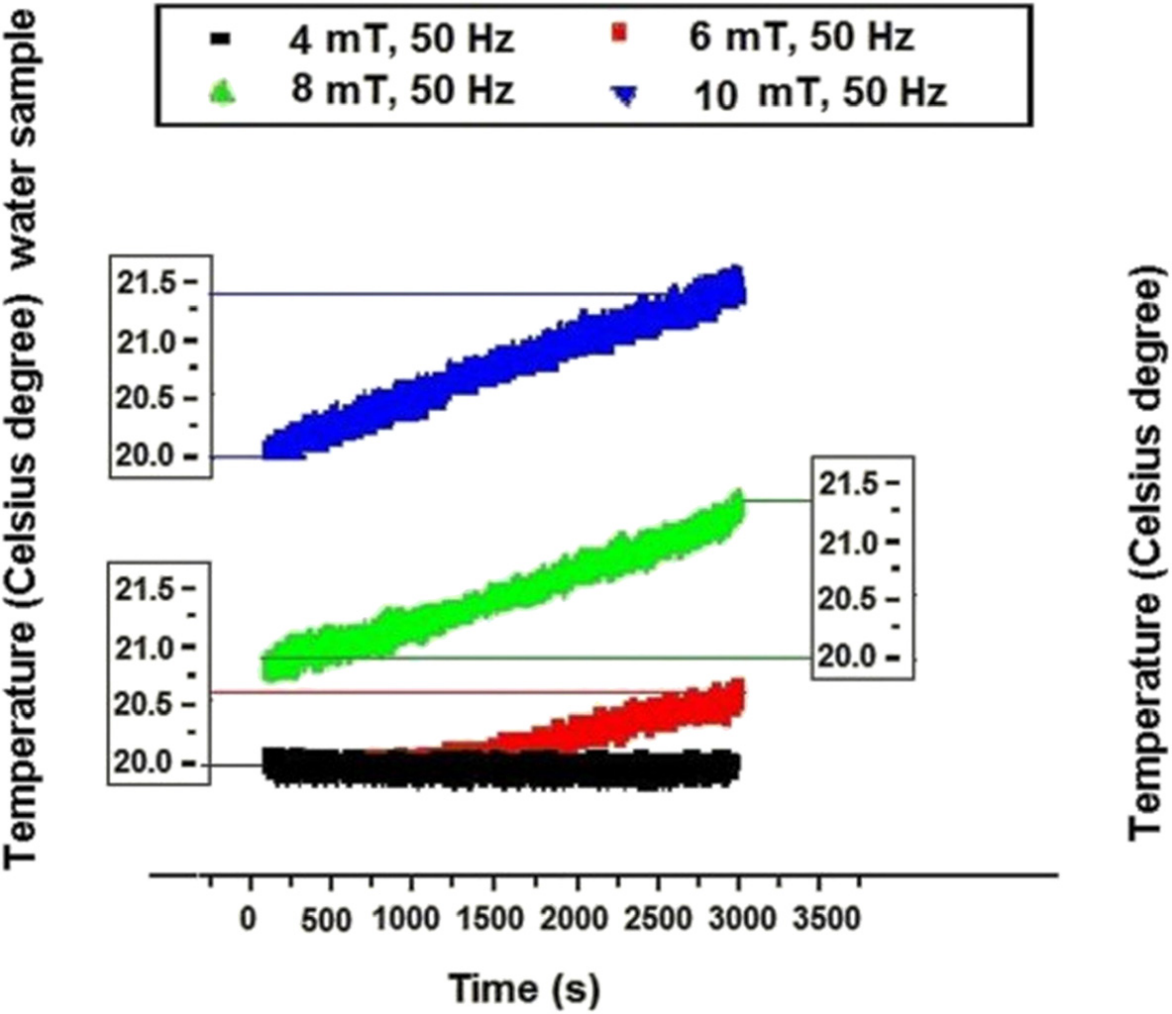
Deionized water electromagnetically exposed (the volume of 2 cm^3^ was considered)

For comparison the deionized water response to the same array of magnetic flux densities is presented in Fig. 9 (the volume of 2 cm^3^ was considered). From Fig. 9 it can be seen that non significant variation was recorded for 4 mT (similar with muscle tissue with highest water content) while for 6 mT the temperature increase with 0.6 °C was noticed and for 8 mT and 10 mT the increase with about 1.3 °C and 1.4 °C respectively, was reached in water.

In theory, when a sample with conductivity *σ* is exposed to a vertical electromagnetic field with frequency *f*, concentric electric current flow occurs, in a plan orthogonal to the direction of the magnetic field. If *r* is the radius of the sample, and *B* is the magnetic flux density in Tesla, then current density of the induced current *J*, is:

1 $$ J=\sigma \cdot \pi \cdot f\cdot B\cdot r $$

The magnetically induced electric field (*E*) does not depend on sample conductivity and is given by:

2 $$ E=\frac{J}{\sigma }=\pi \cdot f\cdot B\cdot r $$

The power (averaged in time) delivered to the sample unit volume is given by:

3 $$ w=\sigma \cdot {E}^2 $$

where *σ* is the sample conductivity and *E* is the magnetically induced electric field in the sample.

Since the investigated tissue samples had around 1 cm^3^ in volume, one can take an approximated value *r* = 1 cm, in order to calculate the maximum of the induced electric field corresponding to the 10 mT magnetic induction. Therefore the maximum of the magnetically induced electric field for B = 10 mT is of about 0.0157 V/m.

Assuming the same dissipation for *r* < 1 cm, the maximum power dissipation per unit volume is: $w=2.46\cdot \sigma \cdot {10}^{-4}\frac{W}{m^3}$. Then for 1 h exposure time the energy delivered to the tissue sample of about 2 cm^3^ is ≈ 0.177 · *σ* · 10^−5^ J. The conductivity (*σ*) for various living tissues in the extremely low frequency magnetic fields is in the range 0.02-1.5 S/m [18].

**Fig. 10:**
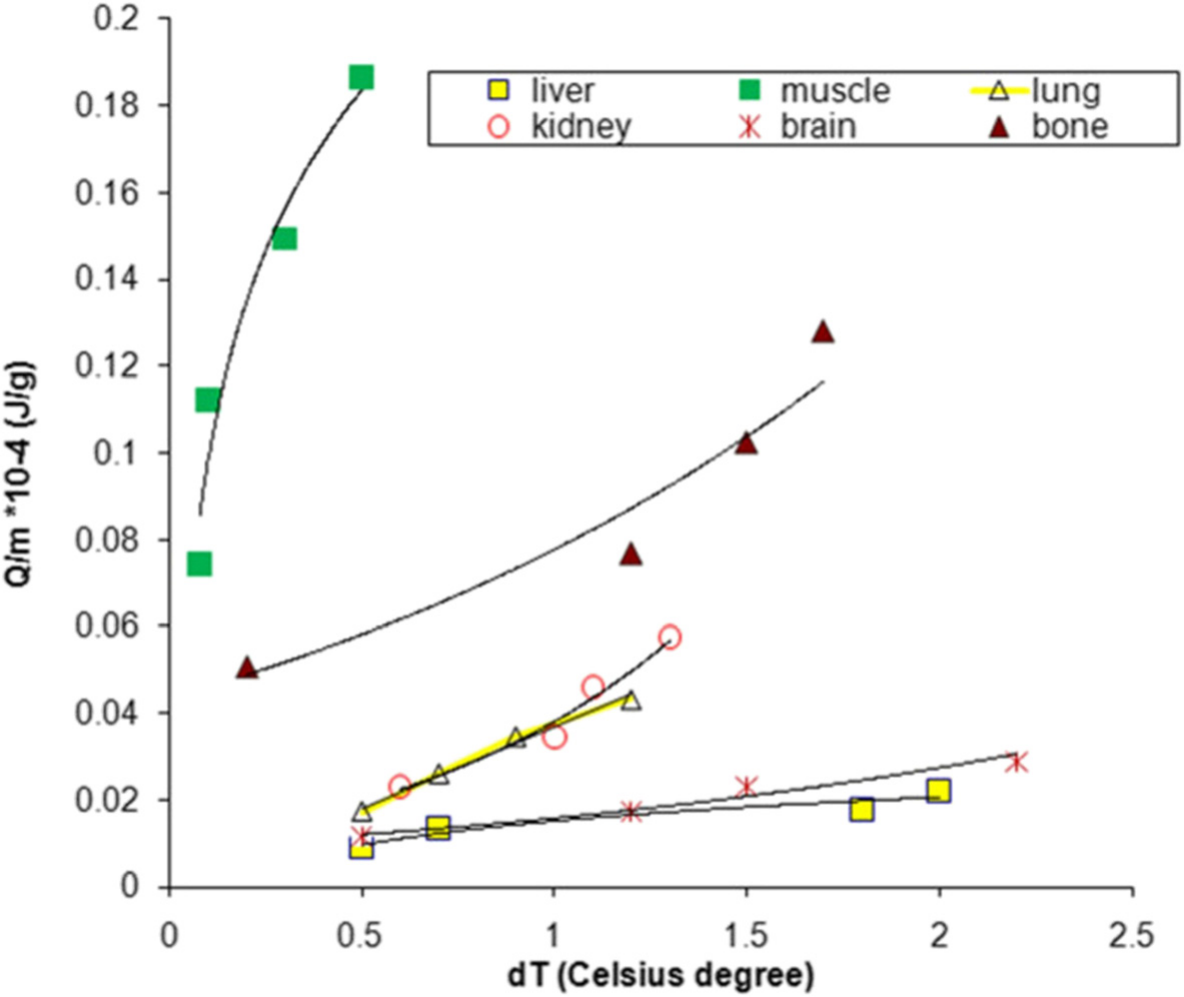
Dependence of delivered electromagnetic energy divided by sample mass (*Q/m*) and temperature

In accord with [18, 19], the electric conductivity values of the tissues at 50 Hz have been used to obtain the energy delivered by electromagnetic field to each sample (*Q*) divided by the mass of tissue sample (*m*). The dependence between these specific energy values on temperature interval corresponding to magnetic flux densities used in this experiment is presented in Fig. 10.

The regression functions fitting of the experimental graphs from Fig. 10 were calculated; thus exponential functions were found to provide best approximation (with highest corresponding correlation coefficient) of experimental curves for kidney, lung, brain and bone tissue and respectively, logarithmic function for liver and muscle tissue (Table 1).

**Table 1:** Regression functions fitting Fig. 10 graphs (*y* represents *Q/m; x* represents *dT*)

Mammalian tissue	Regression function	Correlation coefficient R^2^
liver	*y* = 0.008 ln(*x*) + 0.015	0.92
muscle	*y* = 0.0535 ln(*x*) + 0.2206	0.94
kidney	*y* = 0.0101*e* ^1.3305*x*^	0.97
lung	*y* = 0.0099*e* ^1.2934*x*^	0.95
brain	*y* = 0.0009*e* ^0.5538*x*^	0.97
bone	*y* = 0.0435*e* ^0.5798*x*^	0.94

**Fig. 11:**
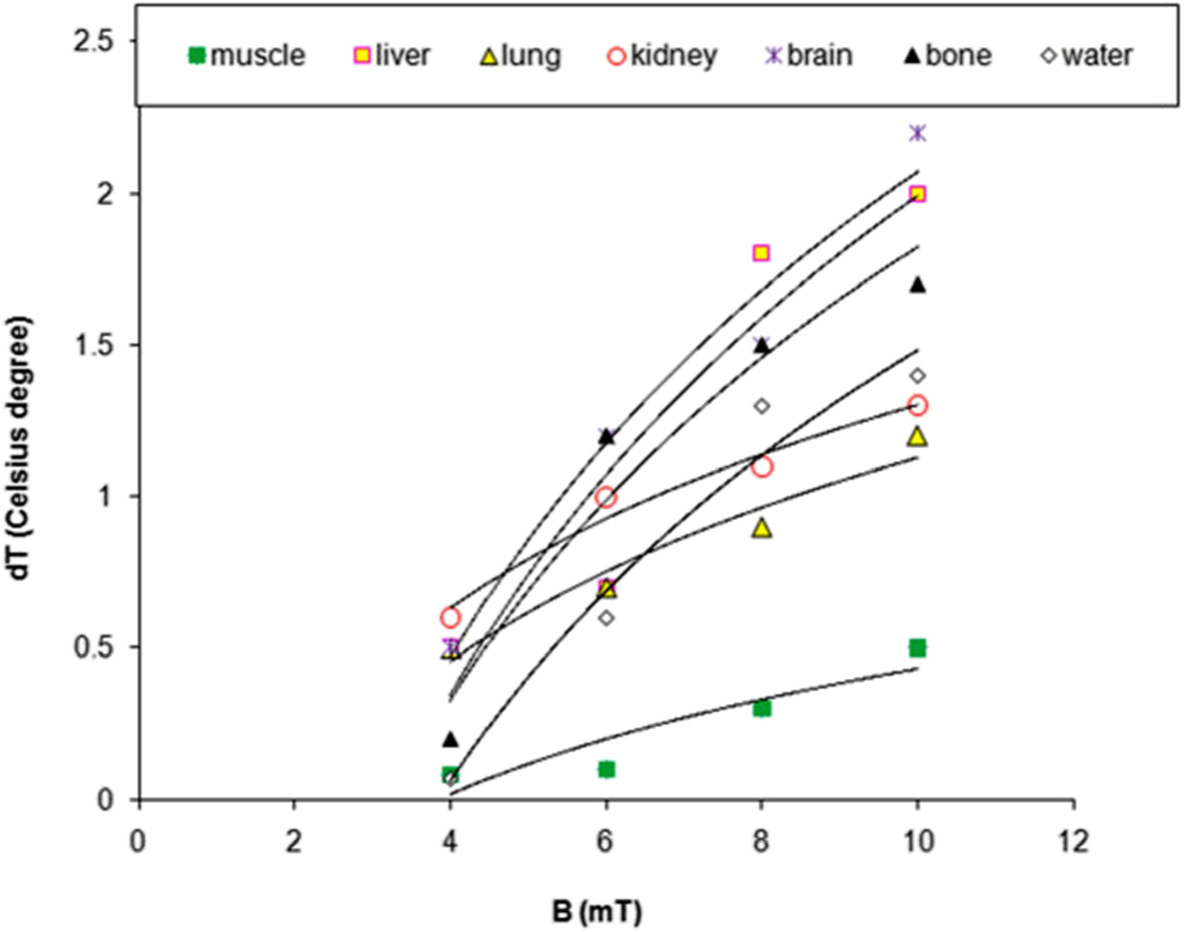
Temperature increase corresponding to magnetic flux density values used in this study

In case of materials with definite specific heat, linear dependences would be evidenced as specific heat is constant. As we obtained obviously non-linear graphs it seems that heating provided by electromagnetic phenomena in complicated biological materials is more complex, with effects on the intimate structure of sample. In Fig. 11 temperature increasing, corresponding to magnetic induction of applied magnetic field is represented. The highest increasing of temperature appears most evident in case of brain tissue for 10 mT magnetic induction; however, as expected, this is lower in the case of water at 4 mT magnetic field exposure (with about 97 %). Bone tissue heating production profile is similar to that of brain tissue for all magnetic field inductions, but it is with about 23 % less “productive” than the first one. In Table 2 are given the logarithmic functions fitting the experimental graphs from Fig. 11 for all tissue types.

**Table 2:** Regression functions fitting graphs from Fig. 11 (*y* represents *dT; x* represents *B*)

Mammalian tissue	Regression function	Correlation coefficient R^2^
liver	*y* = 1.80ln(*x*)-2.10	0.88
muscle	*y* = 0.45ln(*x*)-0.61	0.83
kidney	*y* = 0.73ln(*x*) -0.38	0.97
lung	*y* = 0.73ln(*x*)-0.56	0.95
brain	*y* = 1.75ln(*x*)-1.95	0.97
bone	*y* = 1.6ln(*x*)-1.93	0.94
water	*y* = 1.55ln(*x*)-2.09	0.96

According to above results it seems that similar mechanisms undergo all tissues interaction with electromagnetic radiation, resulting in temperature increasing to the increase of electromagnetic field induction – since the same function type approximates the experimental graphs.

We assume that the heating effect could be the result of Faraday induction, i.e. of electric fields and currents which give raise to charge movement and heat production, that could be further related to electrical parameters of the tissues. When ohmic heating is considered then the current intensity through the coil system is most important, being related to Joule effect. If ohmic heating is the main cause of the temperature dynamics recorded by us then the impedance variation needs also to be considered. Electric measurements evidenced that in time, after tissue sample excision from animal body, the electrical bioimpedance of the organs increases from its *in vivo* level, multiplying twice in a few hours [20]. Related to the above hypothesis of structural changes induced during electromagnetic exposure and reflected in specific heat putative variation we believe that bioimpedance variation is also plausible. Other data were reported in literature discussing tissue changes under the influence of ELF-MF exposure; studies on porcine endothelial cells exposed to 50 Hz electromagnetic field showed the influence on heat shock protein levels and partial relocalization in the nucleus [21]; culture cell electroporation was demonstrated for 10 Hz-10 KHz ELF-MF exposures [22] – which is rather widely spread bioengineering technique, mainly for genetic purposes [23]. Electric conductivity changes in the electroporated cell culture [24–26] using ELF electromagnetic fields suggest that in the animal tissues investigated by us cellular modifications could occur with rather predictable negative consequences on the tissue stability.

Tissue structure and composition could be compromised during exposure to electromagnetic field with detectable thermal effect and with further consequences on the food quality.

Thus, it is possible to hypothesize that temperature increase effect is related both to the heat transfer toward the exposed sample and to the increase in time of excised sample bioimpedance. In the case of water no such variation of impedance could be assumed. But water evaporation during sample exposure could interfere with the other phenomena contributing to the smaller temperature rise recorded for the wet tissue of muscle.

The results presented above could be useful in understanding the electromagnetic sensitivity of human body tissues – much comparable with porcine ones – in the less studied circumstances of low frequency electromagnetic field exposure. Brain heating with about 2° in the vicinity of 50 Hz alternative current leads seems to represent a challenging issue versus the frequent utilization of domestic electrical devices like hair drier, electric shaver device etc. as well as of medical electric apparatus and installations - all supplied from the 50 Hz net (or 60 Hz in oversee countries). For human communities consuming frequently meat and derivatives, the health issues related to fresh food storing in spaces hosting electric lines or devices seems to require more attention to support indirectly prevention of subtle degradation before consuming – especially in public restaurants, student cantinas etc.

Apart from the physical considerations regarding the interaction mechanisms between biological material and electromagnetic field, life scientists and especially nutrition specialists need to pay more attention to biochemical aspects mentioned in introductory paragraph, since enzyme activity changes could trigger energetic balance perturbation with health consequences in extreme hypothetical approach.

## Conclusions

Using a non-perturbing fluoroptic probe device for measuring heating of animal tissues during ELF-MF exposure, the thermal effect was revealed and analyzed. Brain, bone and liver heating dynamics resulted in over 2.0 °C temperature rise compared to the other tissues where lower heating levels were evidenced. Faraday induction and ohmic effect were supposed to be the possible causes of the recorded temperature variation with secondary changes in structural features like cell membrane integrity or tissue bioimpedance. Increased temperature was recorded for increased magnetic flux density. Further research is needed to provide deeper insight in such subtle thermal phenomena that possible affect fresh food during storage even during one hour.
